# Alterations in Gut Microbiota and Metabolic Profiles in Relapsed or Refractory Lymphoma

**DOI:** 10.1002/mbo3.70225

**Published:** 2026-02-02

**Authors:** Yu‐Ying Guo, Kang‐Jing Xue, Liao Wang, Gang‐Gang Wang, Ting‐Ting Zhang, Shu‐Ling Hou

**Affiliations:** ^1^ Department of Lymphoma Oncology, Cancer Center,Shanxi Bethune Hospital Shanxi Academy of Medical Sciences, Third Hospital of Shanxi Medical University, Tongji Shanxi Hospital Taiyuan Shanxi China; ^2^ Third Hospital of Shanxi Medical University, Shanxi Bethune Hospital, Shanxi Academy of Medical Sciences Tongji Shanxi Hospital Taiyuan Shanxi China

**Keywords:** fecal microbiota transplantation, gut microbiota, metabolomics, novel therapeutic targets, R/RL

## Abstract

To identify potential therapeutic strategies for relapsed or refractory lymphoma (R/RL) by examining differences in gut microbiota composition and metabolic profiles between patients with R/RL and those with primary, treatment‐naïve lymphoma (PL), using fecal microbiota analysis and metabolomics. A total of 21 patients with lymphoma were enrolled at the Department of Lymphoma and Oncology, Shanxi Bethune Hospital, between November 2023 and December 2024. The cohort included 14 patients with R/RL and 7 with PL, who served as the control group. Pretreatment fecal samples and clinical data were collected from all participants. Gut microbiota profiling was conducted using 16S rDNA sequencing, including alpha diversity, beta diversity, species composition, and differential abundance. Untargeted metabolomics was employed to identify and analyze differentially expressed metabolites between the groups. Patients with R/RL exhibited increased relative abundances of *Actinobacteriota* and *Alphaproteobacteria* and decreased levels of *Erysipelotrichales*, *Morganellaceae*, *Faecalibacterium*, *Clostridium*, *Klebsiella*, and *Ruminococcus*. Seven metabolites were significantly upregulated in the R/RL group (*p* < 0.05): 3‐amino‐4‐methylpentanoic acid (*p* = 0.028), 2‐hydroxybutyric acid (*p* = 0.020), UDP‐N‐acetylglucosamine (UDP‐N‐AG) (*p* = 0.011), pantothenic acid (*p* = 0.037), isoleucine (*p* = 0.028), glycine (*p* = 0.044), and alanine (*p* = 0.025). Literature review and Kyoto Encyclopedia of Genes and Genomes (KEGG) pathway analysis indicated enhanced central carbon metabolism and amino acid metabolism in cancer. Alterations in gut microbiota and metabolic activity may contribute to the pathophysiology of R/RL. Therapeutic modulation of the gut microbiota, including the use of fecal microbiota transplantation, may improve the intestinal immune microenvironment in this patient population. The present work is hypothesis‐generating and requires large‐scale validation.

## Graphical abstract

Metabolomic volcano‐heatmap reveals seven upregulated metabolites in relapsed/refractory lymphoma, underscoring gut metabolic reprogramming as a potential therapeutic target.

**Abbreviations**
C: class
F: family
G: genus
GM: gut microbiota
M: metabolomics
O: order
P: phylum
PCA: principal co‐ordinates analysis
R/RL: relapsed/refractory diseases
S: species
UDP‐N‐AG: UDP‐N‐acetylglucosamin

## 1 Introduction

Lymphoma represents a highly heterogeneous malignancy with a pathogenesis that remains incompletely understood. Despite considerable advances in the understanding of lymphoma biology and clinical management, effective therapeutic options for patients with relapsed or refractory lymphoma (R/RL) remain limited. The association between oncogenic pathogens and cancer development has been well documented. Mucosa‐associated lymphoid tissue (MALT) lymphoma, a rare extranodal B‐cell tumor, is strongly associated with *Helicobacter pylori* (*H. pylori*) infection, highlighting the potential influence of microbial communities on malignant tumor development and progression. Alterations in the structure, composition, and functional capacity of the gut microbiota may be linked to specific lymphoma subtypes; however, potential links with other lymphoma types require further investigation.

*H. pylori* infection contributes to lymphomagenesis through chronic inflammation, immune evasion, and the induction of genetic alterations. The bacterium promotes malignant transformation of B cells by activating the nuclear factor kappa‐light‐chain‐enhancer of activated B cells (NF‐κB) signaling pathway, inhibiting apoptotic proteins such as B‐cell lymphoma 2 (Bcl‐2), and inducing chromosomal translocations, including t(11;18)(q21;q21) (Salar 2019). In clinical practice, eradication of *H. pylori* has become an established therapeutic strategy in the management of gastric MALT lymphoma. Evidence indicates that butyrate‐producing Eubacterium rectale may inhibit intestinal lymphoma development. Gut microbiota generate numerous metabolites, some of which can influence tumor progression and modulate systemic immune responses. For example, deoxycholic acid (DCA), a prevalent secondary bile acid in humans, has been demonstrated to promote colorectal cancer by inducing DNA damage and disrupting the intestinal mucosal barrier (Cong et al. 2024). Despite these findings, studies investigating the relationship between gut microbiota and R/RL are limited.

The present study examines differences in gut microbiota composition and metabolite profiles between patients with R/RL and those with primary, treatment‐naïve lymphoma (PL). By employing microbiota sequencing and untargeted metabolomics, this study aims to identify potential microbial and metabolic biomarkers or therapeutic targets that may inform future strategies for managing refractory lymphoma.

## 2 Materials and Methods

### 2.1 Study Population

#### 2.1.1 Study Group

Participants were recruited between November 2023 and December 2024 at Shanxi Bethune Hospital, The Third Clinical Medical College of Shanxi Medical University. The study group comprised 14 patients diagnosed with R/RL, while the control group included seven patients with PL. All participants provided written informed consent prior to inclusion. Pathological diagnoses were established according to the 2022 World Health Organization (WHO) Classification of Haematolymphoid Tumors, and lymphoma subtypes were classified based on Han's classification system.

**Inclusion criteria for the R/RL group:** (1) Histologically confirmed diagnosis of malignant lymphoma; (2) failure to achieve at least a PR following four cycles of first‐line immunochemotherapy or two or more cycles of second‐line or subsequent immunochemotherapy; and (3) disease relapse occurring within 12 months following autologous hematopoietic stem cell transplantation (Auto‐HSCT).

**Exclusion criteria for the R/RL group**: (1) History of any prior malignancy.

**Diagnostic criteria for R/RL:** Relapse disease was defined as progressive disease (PD) occurring within 6 months of achieving complete remission (CR). Refractory disease was defined as failure to achieve at least a PR to the most recent chemotherapy regimen or PD occurring within 6 months following the last chemotherapy cycle. Definitions were based on the 2014 Lugano response criteria.

#### 2.1.2 Control Group

**Inclusion criteria for the PL group:** (1) Histologically confirmed diagnosis of lymphoma and (2) no history of chemotherapy or radiotherapy prior to enrollment.

**Exclusion criteria for the PL group:** (1) History of any prior malignancy.

### 2.2 Methods

#### 2.2.1 Clinical Data Collection

Eligible patients were enrolled following confirmation that inclusion and exclusion criteria were met. Pretreatment fecal samples were collected from participants with PL and from those with R/RL at the time of relapse or upon confirmation of refractory disease.

Clinical data were extracted from medical records, including sex, age, pathological diagnosis, subtype, clinical stage, Eastern Cooperative Oncology Group (ECOG) performance status, International Prognostic Index (IPI) score and risk category, treatment regimen, therapeutic response, and follow‐up outcomes. Pathological classification followed the 2022 WHO Classification of Haematolymphoid Tumors (5th edition), with subtype stratification based on Han's classification. Staging followed the Ann Arbor system, and treatment responses were evaluated using the Lugano criteria.

The R/RL cohort included 14 patients: 12 diagnosed with diffuse large B‐cell lymphoma (DLBCL) and 2 with T‐cell non‐Hodgkin lymphoma (T‐NHL). The cohort comprised 10 males and 4 females. Among the patients with DLBCL, ages ranged from 35 to 80 years (median: 52 years). Disease stages were as follows: stage II (*n* = 2), stage III (*n* = 1), and stage IV (*n* = 9). IPI scores were distributed as follows: 0 points (*n* = 1), 2 points (*n* = 4), 3 points (*n* = 4), and 5 points (*n* = 3). ECOG performance status scores were 1 (*n* = 5), 2 (*n* = 4), and 3 (*n* = 3). Risk stratification included low risk (*n* = 1), low‐intermediate risk (*n* = 4), intermediate‐high risk (*n* = 4), and high risk (*n* = 3). Subtype analysis identified four cases of germinal center B‐cell (GCB) origin and eight non‐GCB cases; one patient harbored MYC and Bcl‐2 double‐hit alterations. The two T‐NHL cases comprised angioimmunoblastic T‐cell lymphoma (AITL) and monomorphic epitheliotropic intestinal T‐cell lymphoma (MEITL), both classified as stage IV. Both had IPI scores of 2, with ECOG scores of 2 (*n* = 1) and 3 (*n* = 1), and both were classified as low‐intermediate risk.

The control group included seven patients with PL: four with DLBCL, two with follicular lymphoma (FL), and one with T‐NHL. This group included three males and four females. Among individuals with DLBCL, disease stages were: stage I (*n* = 1), stage II (*n* = 1), stage III (*n* = 1), and stage IV (*n* = 1). IPI scores were 1 (*n* = 2), 2 (*n* = 1), and 5 (*n* = 1). ECOG scores were 0 (*n* = 1), 1 (*n* = 1), 2 (*n* = 1), and 3 (*n* = 1). Risk stratification included low risk (*n* = 2), low‐intermediate risk (*n* = 1), and intermediate‐high risk (*n* = 1). The two FL cases were staged as II (*n* = 1) and III (*n* = 1), with IPI scores of 1 and 2, ECOG scores of 1 and 3, and risk classifications of low (*n* = 1) and high (*n* = 1). The T‐NHL case was stage III, with an IPI score of 1, an ECOG score of 3, and a high‐risk classification.

Both cohorts exhibited comparable age ranges (35–80 years), and no significant differences were observed in sex distribution, disease stage, or ECOG performance status.

Treatment for patients with DLBCL in both cohorts included the standard first‐line R‐CHOP regimen, with or without additional agents. Second‐line therapies included R‐GDP, R‐GemOx, or R‐ICE/R‐DICE with or without anti‐CD79 antibodies. Subsequent treatment options comprised CD19‐CAR‐T cell therapy and bispecific anti‐CD20 antibodies. For patients with T‐NHL, first‐line included CHOP or CHOEP, while second‐line treatments consisted of GDP, GemOx, ICE, or DICE, administered with or without agents such as chidamide, lenalidomide, or golisitinib.

In the control group, five patients achieved partial response (PR) and two achieved CR following initial therapy. No cases of relapsed or refractory disease were observed during 1‐year of follow‐up.

#### 2.2.2 Fecal Sample Collection

Patients were instructed to line the bedpan with clean paper prior to defecation. Approximately 1 g of stool was collected using a sterile spoon and transferred to sterile collection tubes. Samples were immediately stored at −80°C until subsequent analysis.

#### 2.2.3 Fecal 16S rDNA Analysis

##### 2.2.3.1 Microbial DNA Extraction

Total microbial genomic DNA was extracted from fecal samples using a magnetic bead‐based fecal genomic DNA extraction kit. DNA concentration was measured using a Qubit fluorometer (Invitrogen, Carlsbad, CA, USA). The bacterial 16S rRNA V3‐V4 region was amplified by polymerase chain reaction (PCR) using primers 515 F (5′‐barcode‐GTGCCAGCMGCCGCGG‐3′) and 907 R (5′‐CCGTCAATTCMTTTRAGTTT‐3′). PCR conditions were as follows: initial denaturation at 98°C for 30 s, 32 cycles of denaturation at 98°C for 10 s, annealing at 54°C for 30 s, and extension at 72°C for 45 s, followed by a final extension at 72°C for 10 min.

##### 2.2.3.2 PCR Product Quantification

Amplified PCR products were purified using AMPure XP magnetic beads, and DNA concentrations were subsequently determined using a Qubit fluorometer (Invitrogen, Carlsbad, CA, USA).

##### 2.2.3.3 Library Preparation and Sequencing

Library QC was performed using an Agilent 2100 Bioanalyzer in combination with an Illumina library quantification kit. Libraries with concentrations ≥ 2 nM were subjected to gradient dilution, equimolar pooling, and alkaline denaturation prior to sequencing. Paired‐end sequencing (PE250) was conducted on the Illumina NovaSeq. 6000 platform using an SP 500‐cycle reagent kit.

##### 2.2.3.4 Data Processing

**Data demultiplexing:** Raw paired‐end sequencing data were demultiplexed using bcl2fastq, which simultaneously removed sequencing adapters and index sequences.

**Data processing steps included:** Adapter trimming, performed using Cutadapt to remove primer and adapter sequences from FASTQ files. Overlapping paired‐end reads (PE150) were merged into contigs using the PEAR algorithm. Dynamic QC was conducted using a sliding window (100 bp) with a Q20 threshold. Sequences were truncated at the 3′ end when the thresholds were not met. Length filtering reads were retained if ≥ 100 bp in length and contained < 5% ambiguous bases (N). PCR chimeras were identified and removed using VSEARCH with comparison to the UCHIME reference database.

##### 2.2.3.5 DADA2 Denoising

Sequence denoising was performed using the QIIME dada2 denoise‐paired pipeline. This process included length filtering and error correction to minimize sequencing noise. Amplicon sequence variants (ASVs) and corresponding abundance tables were generated, with singleton ASVs excluded from analysis.

##### 2.2.3.6 Diversity Analysis

Alpha and beta diversity analyses were conducted using ASV sequences and abundance matrices. Alpha diversity was assessed within‐sample diversity using the Shannon index and Chao1 richness estimator. Beta diversity analysis employed weighted and unweighted UniFrac distances, Jaccard similarity coefficient, and Bray‐Curtis dissimilarity, followed by statistical testing to determine intergroup differences.

##### 2.2.3.7 Taxonomic Annotation

Species abundance data were used for intergroup differential analysis. For groups with biological replicates, the Kruskal–Wallis test was applied to evaluate intergroup differences, with statistical significance defined as *p* < 0.05.

##### 2.2.3.8 Differential Abundance Analysis

Differential species abundance across groups was analyzed using non‐parametric statistical methods based on species abundance matrices. For non‐normally distributed data sets with biological replicates, the Kruskal–Wallis test was used for multiple‐group comparisons. *p*‐values were adjusted using the Benjamini‐Hochberg method, with adjusted significance set at *p* < 0.05. Analyses followed standard protocols for microbiome differential abundance testing.

#### 2.2.4 Untargeted Metabolomics (LC‐MS/MS)

##### 2.2.4.1 Sample Collection

A total of 21 fecal samples from patients in the R/RL and PL groups were analyzed using untargeted metabolomics. All samples were immediately stored at −80°C upon collection and maintained until further processing.

##### 2.2.4.2 Data Analysis Overview

Comparative statistical analyses were conducted between the PL and R/RL groups. Fold change for each metabolite was calculated as the ratio of quantitative values in the R/RL group relative to those in the PL group.

##### 2.2.4.3 Metabolite Extraction

Fecal samples (25.0 ± 0.5 mg) were combined with 500 μL of pre‐cooled extraction solvent (methanol:acetonitrile:water, 2:2:1, v/v/v, containing isotope‐labeled internal standards) at −40°C. Samples underwent bead‐beating homogenization at 35 Hz for 4 min, followed by ice‐bath sonication for 5 min. This extraction‐sonication cycle was repeated three times. The mixtures were equilibrated at 40°C for 1 h and then centrifuged at 12,000 × g and 4°C for 15 min. A 100 μL aliquot of the supernatant was used for liquid chromatography analysis. Equal volumes of all sample extracts were pooled to prepare quality control (QC) samples for monitoring instrument stability.

##### 2.2.4.4 Instrumental Analysis

Polar metabolites were analyzed using a Vanquish UHPLC system coupled to an Orbitrap Exploris 120 high‐resolution mass spectrometer. Chromatographic separation was performed on a Waters ACQUITY UPLC BEH Amide column (2.1 × 50 mm, 1.7 μm). The mobile phase consisted of (A) aqueous 25 mM ammonium acetate with 25 mM ammonium hydroxide and (B) acetonitrile, applied under binary gradient elution. Autosampler temperature was maintained at 4°C, and the injection volume was 2 μL. Mass spectrometric detection was conducted using the Orbitrap Exploris 120, with instrument control and data acquisition performed via Xcalibur 4.4 software. Data were acquired in full‐scan mode (m/z 70–1050) combined with data‐dependent acquisition (DDA) MS/MS fragmentation.

##### 2.2.4.5 Data Processing

Metabolomics data were processed using a multi‐module bioinformatics framework. Raw mass spectrometry files were converted to mzXML format using ProteoWizard. Metabolite annotation was performed with a custom R‐based metabolomics pipeline integrated with the BiotreeDB V3.07 database, employing dual validation through high‐accuracy mass matching (≤ 5 ppm) and MS/MS spectral library matching. Data visualization and multivariate statistical analyses were conducted using an in‐house R‐Shiny engine, supporting applications such as three‐dimensional principal component analysis and interactive metabolic pathway heatmap construction.

### 2.3 Statistical Analysis

#### 2.3.1 16S Rdna Sequencing Statistical Analysis

All analyses adhered to standard biostatistical practices. Data were presented as mean ± standard error of the mean (SEM). Statistical analyses were performed using GraphPad Prism v9.0. Alpha diversity indices and absolute microbial counts were compared using two‐tailed paired *t*‐tests. Microbial community structural heterogeneity was assessed using the non‐parametric Wilcoxon signed‐rank test. Multiple hypothesis testing correction was applied to ensure robustness of the results. Statistical significance was defined as two‐tailed *p* < 0.05.

#### 2.3.2 Untargeted Metabolomics Statistical Analysis

Metabolomics data were analyzed using a standardized bioinformatics pipeline. Raw mass spectrometry data were preprocessed using ProteoWizard and converted to mzXML format. Metabolite annotation was conducted using an in‐house R‐based bioinformatics framework integrated with the BiotreeDB V3.0 metabolomics database, applying dual validation through exact mass matching (mass tolerance ≤ 5 ppm) and MS/MS spectral interpretation. Data visualization and statistical analyses were performed using a custom R package.

##### 2.3.2.1 Bacterial DNA Extraction and Subsequent Sequencing Analysis of the 16S rRNA Gene

Genomic DNA extraction from fecal specimens was executed utilizing the TGuide S96 Magnetic Fecal DNA Kit (TIANGEN Biotech (Beijing) Co. Ltd) in accordance with the manufacturer's instructions. DNA quality verification and concentration quantification were conducted via electrophoresis on a 1.8% agarose gel, while measurements of nucleic acid concentration and assessments of its purity were performed using a NanoDrop 2000 UV‐Vis spectrophotometer (Thermo Scientific, Wilmington, USA). The complete 16S rRNA gene sequences underwent amplification with primers 27F: AGRGTTTGATYNTGGCTCAG and 1492R: TASGGHTACCTTGTTASGACTT. Sample‐specific PacBio barcode sequences were incorporated in both forward and reverse 16S primers for multiplexed sequencing. The amplification process employed KOD One PCR premix (TOYOBO Life Science) with initial denaturation at 95°C for 2 min, succeeded by 25 cycles consisting of denaturation at 98°C for 10 s, annealing at 55°C for 30 s, and extension at 72°C for 1 min 30 s, concluding with extension at 72°C for 2 min to generate amplified products. Purification of PCR amplicons was achieved using VAHTS TM DNA Clean Beads (Vazyme, Nanjing, China), and quantification utilized the Qubit dsDNA HS Assay Kit with a Qubit 3.0 Fluorometer (Invitrogen, Thermo Fisher Scientific, Oregon, USA). After individual quantification procedures, equivalent amounts of amplicons were combined. The SMRTbell library preparation from amplified DNA utilized the SMRTbell Express Template Prep Kit 2.0. The PacBio Sequel II platform (Beijing Biomarker Technologies Co. Ltd., Beijing, China) performed sequencing of the purified SMRT bell libraries from pooled and barcoded samples using the Sequel II Binding Kit 2.0.

##### 2.3.2.2 Analysis of Sequencing Data

Bioinformatics analysis was conducted using BMKCloud (http://www.biocloud.net/). Raw sequencing data underwent initial processing with SMRT Link software (v8.0) for QC and demultiplexing, producing CCS reads. Assignment of sample‐specific CCS sequences was performed using Lima (v1.7.0) through barcode recognition. Cutadapt (v2.7) enabled primer detection and sequence filtration, removing CCS reads lacking primers or outside the target length range (1200–1650 bp). Chimeric sequences were excluded using the UCHIME algorithm (v8.1), yielding clean reads. Sequence clustering into operational taxonomic units (OTUs) at 97% similarity was executed with USEARCH (v10.0), discarding OTUs detected fewer than two times across samples. ASVs were generated with DADA2 (v1.20.0), removing variants present in fewer than two occurrences overall. Taxonomic classification of OTUs was performed with the QIIME2 naive Bayesian classifier against the SILVA database (v138.1) using a 70% confidence cutoff. Alpha diversity indices were calculated in QIIME2 (v2020.6) and visualized in R. Beta diversity patterns were examined through principal coordinate analysis (PCoA), comparing community composition with unweighted Jaccard, weighted Bray‐Curtis, and weighted UniFrac distances. Group‐level differences in beta diversity were statistically evaluated by permutational multivariate analysis of variance (PERMANOVA). Linear discriminant analysis (LDA) effect size (LEfSe) analysis identified taxa with significant intergroup variation, with a logarithmic LDA score cutoff set at 3.019 to emphasize differences in the gut microbiota. BugBase (https://github.com/knights-lab/BugBase) was employed for bacterial phenotype prediction, while PICRUSt2 (https://huttenhower.sph.harvard.edu/picrust) was used to predict microbial community functions, including Cluster of Orthologous Groups of proteins (COG) and Kyoto Encyclopedia of Genes and Genomes (KEGG) pathways.

##### 2.3.2.3 Metabolomic Sequencing and Analysis

For untargeted metabolomics, fecal samples (100 mg ± 1 mg) were mixed with beads and 500 μL of extraction solution [methanol:acetonitrile:water, 2:2:1 (v/v)]. The extraction solution contained deuterated internal standards. The mixed solutions were vortexed for 30 s. LC‐MS/MS analyses were performed using a UHPLC system (Vanquish, Thermo Fisher Scientific) with a Phenomenex Kinetex C18 (2.1 × 100 mm, 2.6 μm) coupled to an Orbitrap Exploris 120 mass spectrometer (Orbitrap MS, Thermo Fisher Scientific). The mobile phase A was 0.01% acetic acid in water, and the mobile phase B was isopropanol:acetonitrile (1:1 v/v). The auto‐sampler temperature was 4◦C, and the injection volume was 2 μL. The raw data were converted to the mzXML format using ProteoWizard and further processed for peak detection, extraction, alignment, and integration using XChromatography Mass Spectrometry(XCMS). Then the BiotreeDB database was applied for metabolite annotation. Background peaks were removed if the intensity in the procedure blank sample was > 0.3‐fold of that in the biological samples. Human sample metabolomics and mouse sample metabolomics raw data were deposited to the MetaboLights database at EBI (https://www.ebi.ac.uk/metabolights/) under accession numbers MTBLS12275 and MTBLS12276, respectively. The following metabolomics analysis and visualization were performed using the Lims2 cloud platform (https://biotree.lims2.com/) provided by BioTree. Metabolite Orthogonal Partial Least Squares Discriminant Analysis (OPLS‐DA) analysis was performed to visualize discrimination between cases and controls and calculate variable importance in the projection (VIP) values for prioritizing differential metabolites. Metabolite intensity data were log‐transformed and Pareto‐scaled, then modeled with group classification as the *Y* variable. The OPLS‐DA model was constructed with one predictive component and orthogonal components determined by cross‐validation. Its performance was assessed using R2 and Q2 values, validated by permutation testing. Variables with VIP scores > 1.0 were identified as significant contributors to group separation. Differential metabolites were selected using multivariate statistical analysis, where a *p*‐value < 0.05 from the Student's *t*‐test, (Log2FoldChange) > 1 and VIP score > 1 were considered significantly different. Volcano plots were generated using the Lims2 cloud platform, plotting log2 FC on the *x*‐axis and −log10 (*p*‐value) on the *y*‐axis, with significant metabolites highlighted in color. Area radar plots were generated to visualize the top 10 metabolites contributing to group separation, selected based on VIP scores (> 1.0) from OPLS‐DA and *p*‐values (< 0.05). Metabolite intensities were *z*‐score normalized, and plots were constructed using the Lims2 platform. Differential metabolites were annotated with KEGG compound IDs using MetaboAnalyst's mapping tool based on exact mass and compound names prior to enrichment analysis. Enrichment analysis was conducted against the KEGG pathway database; pathways with *p* < 0.05 were considered significantly enriched. Results were visualized as a bubble plot using the Lims2 platform. Spearman correlation analysis was performed using GraphPad Prism.

## 3 Results

### 3.1 Clinical Characteristics

In the R/RL group, DLBCL was the predominant subtype (12/14 cases), with the remaining two cases classified as T‐NHL. Early‐stage disease (stages I–II) was present in 2 of 14 patients, whereas advanced‐stage disease (stages III–IV) was observed in 12 of 14 patients. Performance status as assessed by the ECOG scale was favorable (scores 0–1) in of 14 patients, and unfavorable (scores 2–5) in 5 of 14 patients. Prognostic assessment by the IPI indicated a favorable prognosis in 1 of 14 patients, with 13 of 14 patients classified as having poor prognosis.

In the PL group, DLBCL accounted for four of seven cases, follicular lymphoma (FL) for two of seven cases, and T‐NHL for one of seven cases. Early‐stage disease (stages I–II) was present in one of seven patients, and advanced‐stage disease (stages III–IV) was present in six of seven patients. COG performance status was favorable (scores 0–1) in 4 of 7 patients and unfavorable (scores 2–5) in three of seven patients. IPI‐based prognosis was favorable in four of seven patients and poor in three of seven patients.

Age distribution was comparable across both groups (range: 35–80 years). No statistically significant differences were identified between groups in terms of sex, disease stage, or performance status. Detailed clinical characteristics are presented in Tables 1 and 2.

**Table 1:** Clinical characteristics of patients in the R/RL group.

Sample	Sex	Age	Diagnosis	Ann Arbor	Subtype	IPI	ECOG	Risk stratification	Follow‐up (months)
1	Male	62	DLBCL	IVB	non‐GCB	2	1	Low‐intermediate risk	41
2	Male	61	DLBCL	IVA	GCB	2	1	Low‐intermediate risk	12
3	Male	73	DLBCL	IVB	non‐GCB	5	3	High risk	38
4	Female	37	DLBCL	IVA	non‐GCB	3	2	Intermediate‐high risk	9
5	Female	66	DLBCL	IIA	GCB	3	2	Intermediate‐high risk	20
6	Male	63	DLBCL	IVB	non‐GCB	2	2	Low‐intermediate risk	Death
7	Male	59	DLBCL	IVA	non‐GCB	5	1	High risk	20
8	Female	58	DLBCL	IIA	GCB	0	1	Low risk	53
9	Male	79	DLBCL	IVA	non‐GCB	5	2	High risk	48
10	Male	63	DLBCL	IIIA	GCB	2	1	Low‐intermediate risk	61
11	Male	34	DLBCL	IVB	non‐GCB	3	2	Intermediate‐high risk	16
12	Female	63	DLBCL	IVA	non‐GCB	3	3	Intermediate‐high risk	Death
13	Male	74	T‐NHL	IVB	AITL	2	2	Low‐intermediate risk	82
14	Male	68	T‐NHL	IVA	MEITL	2	3	Low‐intermediate risk	8

**Table 2:** Clinical characteristics of patients in the (treatment‐naïve) PL group.

Sample	Sex	Age	Diagnosis	Ann Arbor stage	IPI	ECOG	Risk stratification	1‐year follow‐up response
1	Male	69	FL grade 3A	IIA	1	1	Low risk	PR
2	Male	83	FL grade 2	IIIA	2	3	Low‐intermediate risk	PR
3	Female	70	Transformed DLBCL	IVA	3	1	Intermediate‐high risk	PR
4	Male	32	PTCL	IIA	1	1	Low risk	CR
5	Female	55	DLBCL(GCB)	IA	1	0	Low risk	CR
6	Female	54	DLBCL(GCB)	IIIB	5	3	High risk	PR
7	Female	70	DLBCL(GCB)	IVA	5	2	High risk	PR

### 3.2 16S RDNA Sequencing Analysis

#### 3.2.1 Alpha Diversity Analysis

**Figure 1:**
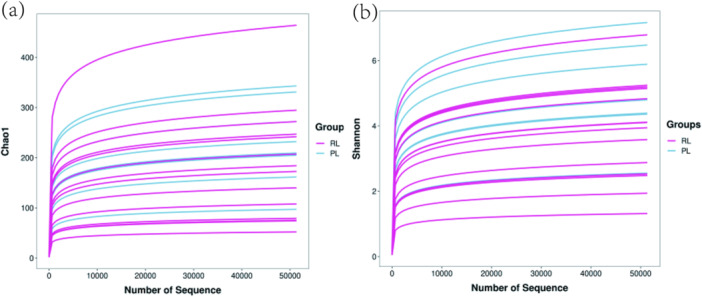
Alpha diversity indices of gut microbiota in PL and R/RL groups. (a) Chao1 richness index between PL and R/RL groups. (b) Shannon diversity index between PL and R/RL groups. No significant differences were observed between groups (*p* > 0.05).

Community richness was estimated using the Chao1 index, while community diversity was assessed using the Shannon index, which reflects information entropy. Higher Shannon values indicate greater diversity. Comparison of gut microbiota between the R/RL and PL groups showed no significant differences in Chao1 richness or Shannon diversity indices (*p* > 0.05) (Figure 1).

#### 3.2.2 Beta Diversity Analysis

**Figure 2:**
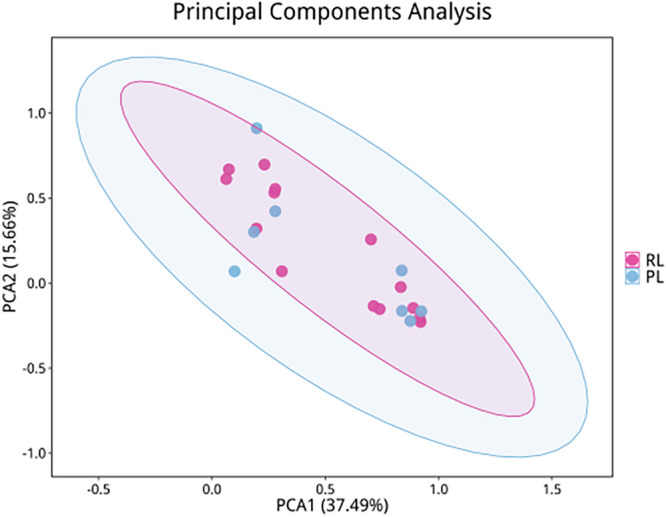
Principal coordinate analysis of microbial community structure. No significant spatial segregation was observed between PL and R/RL groups.

**Figure 3:**
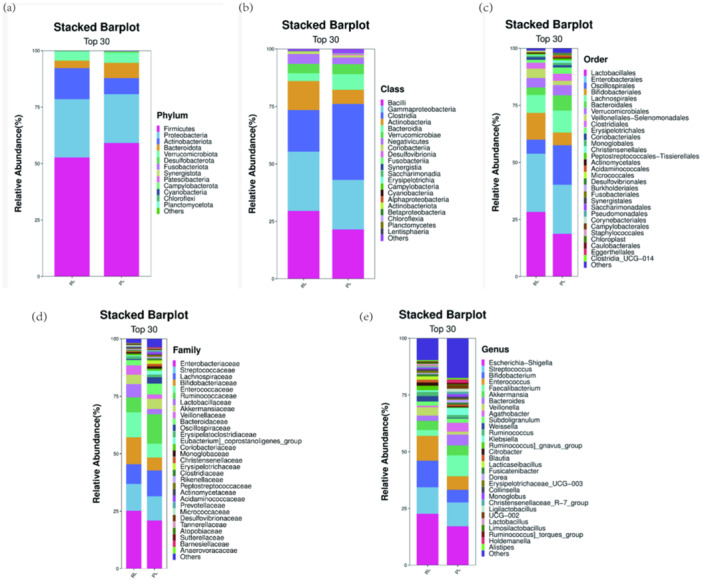
Taxonomic composition of gut microbiota in PL and R/RL groups. Differential distributions are shown at the (a) Phylum, (b) Class, (c) Order, (d) Family, and (e) Genus levels.

PCoA demonstrated no significant differences in overall microbial community structure between groups (*p* > 0.05). In the dimensionality reduction analysis, PC1 and PC2 explained 37.49% and 15.66% of the variance, respectively, with a cumulative contribution of 53.15%. Visualization of score plots indicated no distinct spatial segregation between the R/RL and PL groups (Figures 2 and 3).

#### 3.2.3 Taxonomic Composition Analysis

Phylogenetic hierarchical analysis indicated no significant differences in taxonomic distribution from phylum to species levels (*p* > 0.05). However, several taxa demonstrated significant differential abundance (*p* < 0.05) at specific taxonomic levels.

Phylum level (Figure 3a): Increased abundance of *Actinobacteriota* in R/RL, with reduced abundances of *Firmicutes* and *Bacteroidota*.

Class level (Figure 3b): Increased abundance of *Bacilli* and *Actinobacteria* in R/RL, with reduced abundances of *Clostridia*.

Order level (Figure 3c): Increased abundance of *Bifidobacteriales*, with reduced abundances of *Oscillospirales*.

Family level (Figure 3d): Increased abundance of *Enterobacteriaceae*, *Eubacteriaceae*, and *Bifidobacteriaceae*, with reduced abundances of *Lactobacillaceae*.

Genus level (Figure 3e): Increased abundance of *Escherichia‐Shigella*, *Enterococcus*, and *Bifidobacterium*, with reduced abundances of *Faecalibacterium* and *Klebsiella* in R/RL.

#### 3.2.4 Venn Diagram Analysis of Species Composition

**Figure 4:**
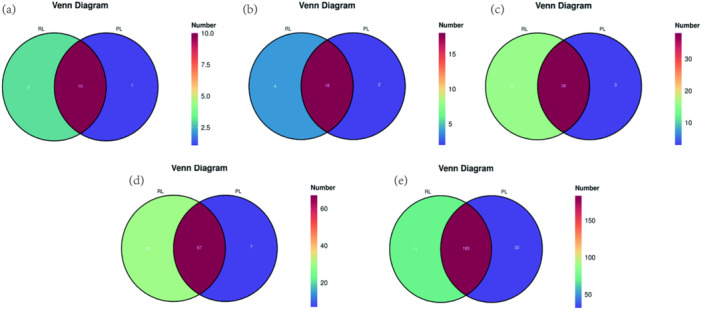
Venn diagrams of shared and unique microbial taxa in PL and R/RL groups. Taxa overlap and uniqueness are displayed at the (a) phylum, (b) class, (c) family, (d) order, and (e) genus levels.

Venn diagrams were constructed to visualize shared and unique taxa across taxonomic levels. At the phylum level, 10 phyla were shared between groups, with three unique to the R/RL group and one unique to the PL group (Figure 4a). At the class level, 18 classes were shared, with four unique to the R/RL group and two to the PL group (Figure 4b). At the family level, 38 families were shared, with 16 unique to the R/RL group and 3 to the PL group (Figure 4c). At the order level, 67 orders were shared, with 30 unique to the R/RL group, and 7 to the PL group (Figure 4d). At the genus level, 183 genera were shared, with 71 unique to the R/RL group and 32 to the PL group (Figure 4e).

#### 3.2.5 Differential Abundance Analysis

**Figure 5:**
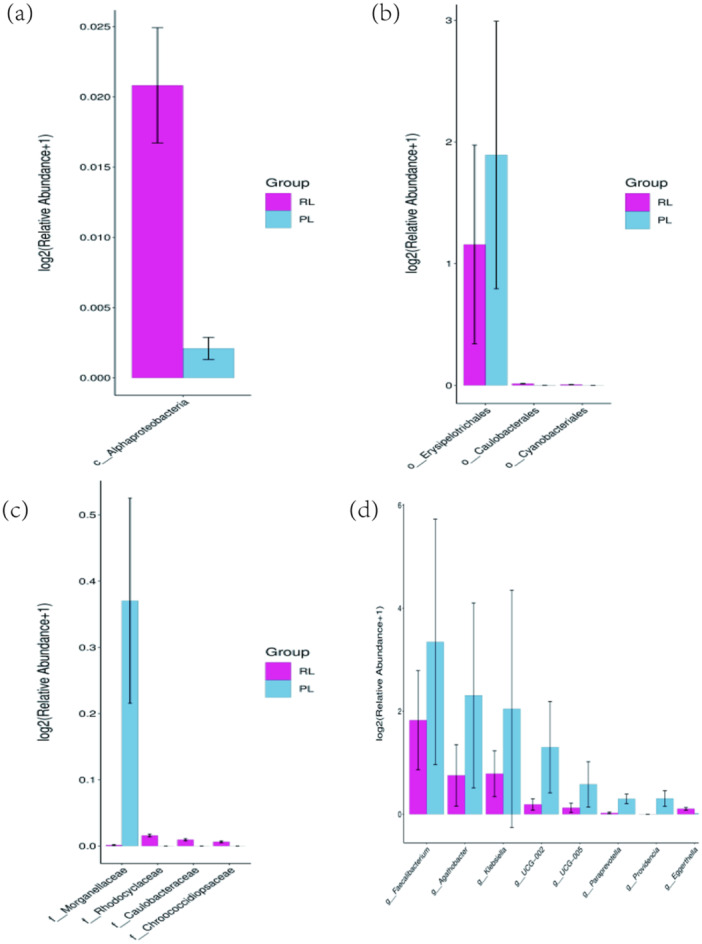
Differentially abundant bacterial taxa between PL and R/RL groups. Significant differences are shown at the (a) class, (b) order, (c) family, and (d) genus levels.

Significant differences were observed across class, order, family, and genus levels. Class level (Figure 5a): *Alphaproteobacteria* increased in the R/RL group (*p* < 0.05). Order level (Figure 5b): *Erysipelotrichales* decreased in the R/RL group (*p* < 0.05). Family level (Figure 5c): *Morganellaceae* decreased in the R/RL group (*p* < 0.05). Genus level (Figure 5d): *Faecalibacterium*, *Clostridium* (Agathobacter), *Klebsiella*, *Ruminococcus* (UCG‐005), and UCG‐002 were all significantly reduced in the R/RL group (*p* < 0.05).

### 3.3 Untargeted Metabolite Analysis

#### 3.3.1 Screening of Differential Metabolites

In mass spectrometry analysis, each metabolite was characterized by a unique combination of its mass‐to‐charge ratio (m/z) and retention time (RT). Relative metabolite abundances were quantified by integrating chromatographic peak areas, thereby enabling comparison of metabolite levels across samples. Instrument performance was evaluated using internal standards in QC samples. Extracted ion chromatogram (EIC) analysis indicated that the relative standard deviation (RSD) of RTs for QC internal standards remained within 2%, while the RSD of signal intensity was maintained below 10%. These results demonstrated high reproducibility and stability of the detection system throughout the analytical sequence.

**Figure 6:**
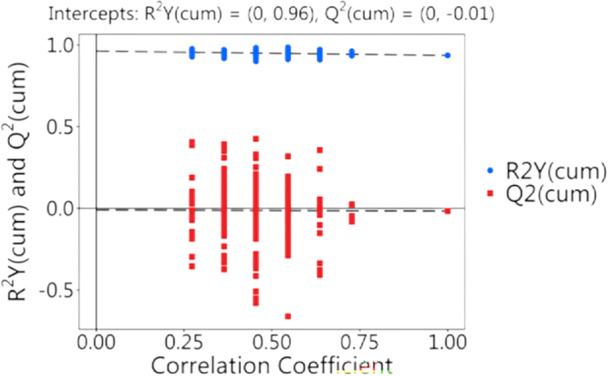
Permutation test validation of the OPLS‐DA model. Comparison of model parameters (*R*², *Q*²) between original and permuted models for PL versus R/RL groups.

Model validation was conducted using permutation testing of the OPLS‐DA model. Two hundred random permutations of sample class labels were generated with model reconstruction, and the explained variance (*R* ^2^) and predictive ability (*Q* ^2^) were calculated for each iteration. Comparison of permuted distributions with original model indices was used to assess overfitting risk and establish statistical validity, following standard multivariate validation procedures. The validation results, presented as score and permutation plots, demonstrated *R*² and *Q*² values of 0.096 and 0.001, respectively. The *R*²–*Q*² difference was less than 0.3, confirming the absence of overfitting and supporting model robustness. Although the low Q² value reflected limited predictive performance, the model captured clear group separation patterns (VIP > 1.0), indicating potential utility in biomarker identification. Permutation test results for the OPLS‐DA model comparing the PL and R/RL groups are presented in Figure 6.

**Figure 7:**
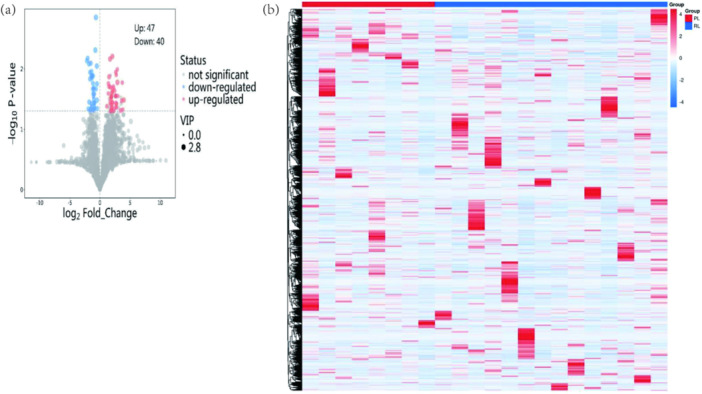
Metabolomic profiling of differential metabolites between PL and R/RL groups. (a) Volcano plot illustrating significantly upregulated and downregulated metabolites. (b) Hierarchical clustering heatmap of differential metabolites.

Differential metabolites were identified using a dual screening strategy that combined Student's *t*‐test (*p* < 0.05) with variable importance in projection (VIP) scores derived from the OPLS‐DA model (VIP ≥ 1). This approach incorporated both statistical significance and variable contribution to model classification, thereby enhancing result reliability. A total of 87 differential metabolites were detected, with 47 upregulated and 40 downregulated. Normalized data visualized using a volcano plot (Figure 7a) and a heatmap (Figure 7b) demonstrated distinct metabolic differences between groups. Significantly upregulated metabolites in the R/RL group included 3‐amino‐4‐methylpentanoic acid, 2‐hydroxybutyric acid, UDP‐N‐acetylglucosamine (UDP‐N‐AG), pantothenic acid, isoleucine, glycine, and alanine.

#### 3.3.2 KEGG Enrichment Analysis of Differential Metabolites

**Figure 8:**
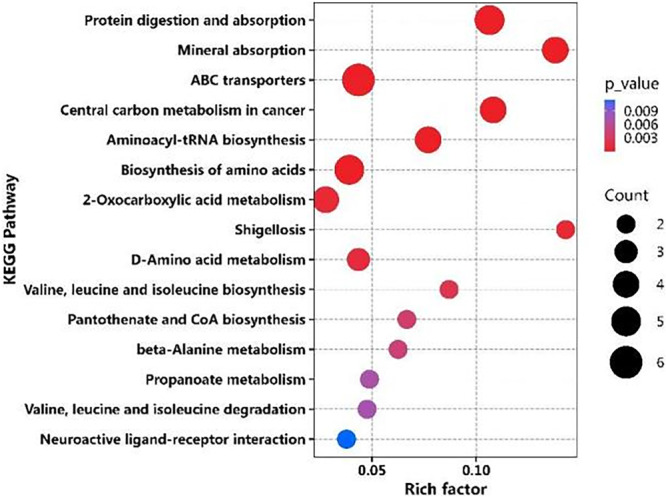
KEGG pathway enrichment analysis of differential metabolites between PL and R/RL groups.

Pathway enrichment analysis was performed using the KEGG database to evaluate the biological relevance of differential metabolites. Enrichment intensity was quantified using the rich factor, defined as the ratio of differential metabolites to the total number of reference metabolites in a given pathway. Higher rich factor values indicated stronger pathway involvement. Multiple hypothesis testing correction was applied using the Benjamini‐Hochberg method, with statistical significance defined as a false discovery rate (FDR)‐adjusted *Q*‐value > 0.05. The KEGG enrichment analysis (Figure 8) demonstrated significant enrichment in pathways including protein digestion and absorption, mineral absorption, ABC transporters, central carbon metabolism in cancer, aminoacyl‐tRNA biosynthesis, and 2‐oxobutanoate metabolism.

## 4 Discussion

According to their different physiological functions, the human intestinal flora can be divided into conditional pathogenic bacteria (such as Enterobacteriaceae and Enterococcus) and symbiotic bacteria (such as Lactobacillus, Bacteroides fragilis, and Bifidobacterium). For healthy adults, the composition of the intestinal flora is relatively stable. A number of studies have confirmed that the intestinal flora is inextricably linked to the occurrence and development of obesity, diabetes, metabolic syndrome, digestive system diseases, and a variety of vascular diseases (including coronary heart disease, pulmonary hypertension, cerebrovascular diseases, and peripheral vascular diseases). It has been reported that the increased abundance ratio of Firmicutes/Bacteroidetes bacteria may be related to the onset of coronary heart disease, and intestinal flora imbalance may be related to advanced coronary heart disease (Toya et al. 2020). The occurrence of hepatocellular carcinoma (HCC) and colon cancer is related to the increase of pro‐inflammatory bacteria and the decrease of beneficial bacteria (Ponziani et al. 2019). Butyrate‐producing strains were significantly reduced in patients with NK/T‐NHL, and the strains could reduce tumor burden and inhibit tumor development (Shi et al. 2025). At present, the research on intestinal flora and tumors is booming. There are a few studies on lymphoma and intestinal flora.

In this study of the intestinal flora of R/RL patients after chemotherapy, it was found that Actinobacteriota (p‐Actinobacteriota) and Alphaproteobacteria (c‐Alphaproteobacteria) were dominant in R/RL. The abundance of Actinobacteriota in healthy people was only 0.447%, and the abundance of Proteobacteria was 2.747%. Actinomycetes are Gram‐positive and mostly anaerobic or microaerophilic. Actinomycetes are conditional pathogens and also normal flora in the human body. They colonize in the oral cavity, gastrointestinal tract, respiratory tract, and other parts and play a certain role in maintaining the microecological balance of the human body. Bacteria of the phylum Proteobacteria are all Gram‐negative bacteria. There are two taxonomic groups in α‐Proteobacteria, Chlamydia and Rickettsia, which are obligate intracellular pathogens. It can be seen that the dominant flora of relapsed lymphoma patients is different from that of untreated lymphoma patients and healthy people. Conditional pathogens are dominant, while beneficial bacteria of the phylum Firmicutes and Bacteroidetes are reduced. This is different from the dominant intestinal flora of coronary heart disease, liver cancer, and colon cancer. The results are consistent with the study of microbial diversity and composition in patients with R/R multiple myeloma in CR and PR.

Compared with newly diagnosed lymphoma patients, R/RL patients have lower immune function. It has been reported that the intestinal flora of lymphoma patients changed before and after treatment, and the Akkermansia genus decreased after treatment (Merryman et al. 2024). Akkermansia can improve the tumor immune effect. Oral administration of Akkermansia can increase the infiltration of CD4 + T lymphocytes into the tumor through IL‐12 dependence and restore the anti‐tumor effect of PD‐1 immune inhibitors (Xin et al. 2024). Therefore, the decrease of Akkermansia may be related to the poor prognosis of lymphoma.

This study did not find a decrease in Akkermansia in R/RL, and no relevant studies on the efficacy of PD‐1 immune checkpoint inhibitors were conducted. The sample size needs to be expanded, and further research is needed.

In this study, 16srDNA sequencing of intestinal flora in patients with different R/RL subtypes of lymphoma revealed that the abundance of Faecalibacterium, Clostridium, and Ruminococcus was significantly reduced in R/RL patients compared with fecal samples from patients with newly diagnosed lymphoma. These bacteria are important butyrate‐producing flora. Butyrate mainly enters and exits the inner and outer sides of the cell membrane through the channel protein with monocarboxylate transporter 1 (MCT1) as the carrier (Salvi and Cowles 2021). Sodium‐monocarboxylate transporter 1 (SMCT1) is the second most important carrier channel protein. These two channels stabilize the concentration of butyrate in the cell, thereby inhibiting the regulatory genes related to the cancer cell cycle and reducing the occurrence of tumors (Daly et al. 2005). Butyrate exhibits dual effects of anti‐inflammation and intestinal mucosal protection. The decrease in its concentration may lead to an imbalance in the intestinal microenvironment, thereby promoting the deterioration and development of lymphoma. Butyrate inhibits histone deacetylase (HDAC), increases histone H3K27 acetylation (Shi et al. 2025), and promotes the expression of SOCS1 (a negative regulator of the JAK‐STAT pathway), thereby inhibiting the activation of the JAK‐STAT signaling pathway and reducing the occurrence of lymphoma. Butyrate can inhibit tumor growth by regulating the expression of immunosuppressive markers, especially in gastric cancer, by inhibiting PD‐L1 and IL‐10 expression (Lee et al. 2024). In another study, butyrate can activate CD8+ cells, inhibit the growth of colorectal cancer cells, and enhance the efficacy of PD‐1 inhibitors. By binding to Toll‐like receptor 5 on CD8+ cells, it can activate NF‐κB signaling and induce cytotoxic reactions. These results show that butyrate‐producing flora can play an inhibitory role in various tumors through different mechanisms (Kang et al. 2023). This study suggests that the significant reduction of butyrate‐producing bacteria in R/RL patients may be one of the reasons why lymphoma is more difficult to control. Further cell and animal‐level verification is still needed.

The gut microbiota metabolism plays a role in the occurrence and development of tumors by promoting inflammation and the formation of the tumor microenvironment. The following metabolites were significantly upregulated in the fecal samples of R/RL patients in this study (see Table 3): UDP‐N‐acetylglucosamine, a product of sugar metabolism; isoleucine, glycine, and alanine, three amino acid metabolites; 3‐amino‐4‐methylpentanoic acid, which is associated with specific biosynthetic pathways, such as the synthesis of certain amino acids or alkaloids; 2‐hydroxybutyric acid, which is not a direct metabolite of fat but is closely related to fat metabolism; and pantothenic acid, an important component of sugar metabolism, also known as vitamin B5 or nicotinamide, which is coenzyme A (CoA) produced during the aerobic oxidation of sugar.

**Table 3:** Top seven significantly upregulated differential metabolites in patients with R/RL compared with PL.

MS2 name	VIP	*p* value	*Q*‐value
3‐Amino‐4‐methylpentanoic acid	1.7	0.028	0.76
2‐Hydroxybutyric acid	1.4	0.02	0.76
UDP‐N‐acetylglucosamine	1.5	0.011	0.76
Pantothenic acid	2.1	0.037	0.76
Isoleucine	1.7	0.028	0.76
Glycine	1	0.044	0.76
Alanine	1	0.025	0.76

Further KEGG analysis found that intestinal metabolites were enriched in three pathways: carbon metabolism in cancer, aminoacyl‐tRNA metabolism, and 2‐oxobutanoate metabolism. Carbon metabolism in cancer center includes aerobic glycolysis, tricarboxylic acid cycle disorder, pentose phosphate pathway, and increased glutamine decomposition. UDP‐N‐acetylglucosamine participates in sugar metabolism and plays a role in the occurrence and development of R/RL. Aminoacyl‐tRNA metabolism is a key link in the process of protein synthesis. 2‐Oxobutyric acid, also known as 2‐ketobutyric acid, is a product of cystathionine cleavage and one of the degradation products of threonine, which is deaminated by threonine deaminase. The 2‐oxobutyric acid pathway is mainly related to protein metabolism disorders. It can be seen that aminoacyl‐tRNA metabolism and 2‐oxobutanoate metabolism are the main protein metabolism pathways. Glycine, isoleucine, and alanine are essential amino acids for protein synthesis. Therefore, it is speculated that glycine, isoleucine, and alanine, which are involved in protein metabolism, play a role in the occurrence and development of R/RL. UDP‐N‐acetylglucosamine, glycine, isoleucine, and alanine accumulate in the intestine and affect the sugar and protein metabolism of the intestinal flora, which plays a role in the occurrence and development of R/RL.

The hexosamine biosynthesis pathway (HBP) is a glucose metabolic pathway that synthesizes UDP‐N‐acetylglucosamine for post‐translational modification of intracellular proteins (O‐GlcNAcylation) (Pham et al. 2016). O‐GlcNAc modification participates in cancer biology by regulating transcription factors. Studies in DLBCL have shown that by reducing the level of GlcNAc modification, the nuclear expression of NF‐κB‐p65 and NFATc1 is inhibited, and cell viability is reduced, thereby inhibiting the occurrence and development of lymphoma (Pham et al. 2016). In this study, the level of UDP‐N‐acetylglucosamine in the feces of R/RL patients was significantly higher than that of newly diagnosed lymphoma patients, which indirectly promoted the nuclear expression of NF‐κB‐p65 and NFATc1 (Pham et al. 2016) and further promoted the occurrence of lymphoma. It is speculated that the accumulation of this sugar metabolite may be related to drug resistance and refractory lymphoma. Choueiry et al. found that resistant clones underwent metabolic reprogramming of oxidative phosphorylation during the development of resistance to Bruton's tyrosine kinase (BTK) inhibitors in DLBCL patients (Choueiry et al. 2021), which supports the results of this paper.

Metabolic reprogramming of the serine‐glycine synthesis pathway drives the cellular metabolic pattern to supply one‐carbon metabolic units by activating the endogenous de novo synthesis mechanism. This abnormal regulation suggests that tumor cells may maintain the metabolic demand of nucleotide biosynthesis by strengthening the serine‐glycine axis. Copy number amplification of serine and glycine synthesis genes, as well as genetic alterations in common oncogenes and tumor suppressor genes, can enhance glycine synthesis (Geeraerts et al. 2021), leading to the massive production and secretion of these metabolites that support tumorigenesis. RZ‐2994 (SHIN1) and SHIN2, as dual SHMT1/2 inhibitors, have shown efficacy in DLBCL with glycine uptake defects (Ducker et al. 2017; García‐Cañaveras et al. 2021).

The present study showed that glycine was elevated in R/RL, but serine was not, supporting the view that immune escape leads to the occurrence and development of lymphoma. It can be seen that regulating the metabolic level of glycine may restore immunity and has certain therapeutic value for refractory lymphoma.

Experiments have confirmed that the uptake of alanine by lymphoma cells can promote glycolysis and cell proliferation, and the decrease of serum alanine level plays a key role in the diagnosis and prognosis of ocular adnexal lymphoma (OAL) (Huang et al. 2025). This study found that the elevated level of alanine in the intestine was associated with R/RL, which was related to the excessive uptake of alanine from the intestine by DLBCL cells, promoting glycolysis‐induced drug resistance and then promoting the proliferation of DLBCL cells.

Isoleucine is an essential amino acid and one of the branched‐chain amino acids (BCAAs). BCAAs include Leucine, Isoleucine, and Valine, which are essential amino acids for the human body and must be obtained from food. The gut microbiota also plays an important role in the metabolism of isoleucine. Abnormal BCAAs metabolism may promote the growth, invasion, and metastasis of tumors by activating the mTORC1 signaling pathway, promoting protein synthesis, and providing energy (Wang et al. 2025).

In yellow‐feathered broilers, isoleucine improves intestinal health by regulating the expression of amino acid transporters and genes related to protein metabolism (Ruan et al. 2023). BCAAs are involved in the metabolism of solid tumor liver cancer, melanoma cells (Sheen et al. 2011), and breast cancer (Zhang and Han 2017), but there are no related reports in lymphoma. In this study, intestinal isoleucine in R/RL was significantly upregulated, which needs to be further related to the mechanism of refractory/drug resistance of lymphoma.

It has been reported that elevated serum triglyceride levels and decreased HDL levels are independent prognostic factors for shortened OS in DLBCL patients (Hong et al. 2025). This study found that the intestinal 2‐hydroxybutyric acid was elevated in R/RL patients, which may be related to abnormal lipid metabolism and needs further study. Therefore, it is speculated that intestinal carbohydrate and protein metabolism disorders play an important role in the occurrence and development of R/RL, and the role of lipid metabolism is uncertain.

In summary, intestinal flora imbalance and metabolomics abnormalities in the intestinal microenvironment, mainly including sugar and amino acid metabolism disorders, play a role in the occurrence and development of R/RL. The relationship between the two metabolic disorders of intestinal carbohydrates and proteomics in the occurrence of R/RL needs to be further studied. Multi‐omics studies, including metabolomics, genomics, and proteomics, are underway (Zhang et al. 2025). The key features of tumor metabolic reprogramming include the Warburg effect (aerobic glycolysis) and changes in amino acid metabolism. The metabolic reprogramming of intestinal flora in lymphoma needs to be further studied. A study of DLBCL patients using UPLC‐MS/MS technology found that serum alanine, isoleucine, and so forth, were on the rise compared with the normal control group (*p* < 0.05), while glycine, lysine, and so forth, were on the decline (Zhang et al. 2022). The relationship between the intestinal microenvironment and serum metabolomics changes needs to be further studied to evaluate the role of metabolic microenvironment levels in different parts of lymphoma development.

Although the absence of true healthy controls limits the interpretive depth of our findings, untreated primary lymphoma (PL) patients constitute the most clinically relevant baseline currently attainable. First, ethical considerations preclude collecting pretreatment fecal samples from healthy individuals in most oncology centers; by contrast, PL patients routinely undergo diagnostic colonoscopy or staging PET‐CT, during which surplus stool can be ethically banked. Second, any divergence in the gut ecosystem between PL and R/RL groups is, by definition, acquired after initial chemotherapy and/or clonal evolution toward refractoriness. In other words, the PL profile already embeds the “lymphoma‐intrinsic” microbial and metabolic signature; thus, the incremental shift observed in R/RL patients reflects the superimposed pressure of cytotoxic drugs, antibiotic exposure, stem‐cell transplantation, and immune escape. This paired design is therefore analogous to an “own‐control” comparison over the disease trajectory, minimizing inter‐individual genetic and dietary variance that would be introduced by an external healthy cohort. Consistent with this view, Fenneman AC (Fenneman 2023) employed an identical PL‐baseline strategy, lending external validity to our approach. Nevertheless, we acknowledge that therapy‐naïve PL samples cannot dissociate tumor‐driven from host‐driven alterations; a three‐arm study (healthy + PL + R/RL) will be reported separately.

A second caveat is that our case–control design cannot definitively partition observed alterations into “lymphoma‐intrinsic” versus “therapy‐induced” components. Because all R/RL subjects had received multiple lines of immunochemotherapy, the contracted abundance of butyrate producers such as Faecalibacterium and Ruminococcus may reflect both clonal selection pressure and cytotoxic drug‐associated mucosal injury rather than refractoriness per se. Likewise, the elevated fecal levels of UDP‐N‐acetylglucosamine, glycine, and alanine could stem from tumor‐cell metabolic reprogramming, chemotherapy‐driven mucosal inflammation, or antibiotic‐mediated collateral effects (Table 2). To disentangle these possibilities, we have launched a prospective three‐arm study (healthy donors, treatment‐naïve PL, and matched R/RL) that will profile the gut microbiome and metabolome before, during, and after each therapeutic intervention; the findings will be reported separately and should clarify which signatures are truly specific to the refractory state.

Our cohort unavoidably enrolled a heterogeneous mix of WHO‐defined lymphoma subtypes (DLBCL, FL, AITL, MEITL). Because relapsed/refractory cases are rare and consecutive sampling was used, the distribution of these subtypes differed between the R/RL and PL groups. Different entities vary in biology, prior therapy exposure, and immune micro‐environment, any of which could independently influence gut microbiota or metabolite levels. The present sample size precluded subtype‐matched or subtype‐stratified analyses, so residual confounding by histology cannot be excluded. Multicenter studies with larger, balanced subtype representation are therefore needed to confirm whether the observed microbial and metabolic signatures are truly linked to refractory disease biology rather than to underlying histological diversity.

## 5 Conclusion

Eubacterium, Lactobacillus, and Streptococcus were positively correlated with the response to anti‐PD‐1/PD‐L1 therapy in various types of gastrointestinal cancer. Gut microbiota dysbiosis plays a key role in CAR‐T cell therapy (Stein‐Thoeringer et al. 2023). Improving the intestinal immune microenvironment through fecal microbiota transplantation or probiotic supplementation may reverse tumor resistance and improve the sensitivity of lymphoma to immunochemotherapy and radiotherapy. The present work is hypothesis‐generating and requires large‐scale validation. The mechanism of amino acid metabolism and sugar metabolism in the occurrence and development of lymphoma needs further study. In the future, targeted drugs that regulate the metabolism of amino acids and sugars may reverse the drug resistance of refractory lymphoma and play a role in improving the cure rate of lymphoma.

## Author Contributions

**Yu‐Ying Guo:** conceptualization, methodology, data curation, statistical analysis, writing – original draft. **Kang‐Jing Xue:** conceptualization, methodology, data curation, formal analysis, writing – original draft. **Liao Wang:** data curation, statistical analysis. **Gang‐Gang Wang:** data curation, statistical analysis. **Ting‐Ting Zhang:** data curation, statistical analysis. **Shu‐Ling Hou:** conceptualization, formal analysis, statistical analysis, writing – review and editing, supervision. All authors read and approved the final manuscript.

## Ethics Statement

This study was conducted with approval from the Ethics Committee of Shanxi Bethune Hospital (IIT‐2025‐096‐KS032). This study was conducted in accordance with the Declaration of Helsinki. Written informed consent was obtained from all participants.

## Conflicts of Interest

The authors declare no conflicts of interest.

## Data Availability

All relevant data are within the paper.
